# Fishing for Targets of Alien Metabolites: A Novel Peroxisome Proliferator-Activated Receptor (PPAR) Agonist from a Marine Pest

**DOI:** 10.3390/md16110431

**Published:** 2018-11-03

**Authors:** Rosa Maria Vitale, Enrico D’Aniello, Stefania Gorbi, Andrea Martella, Cristoforo Silvestri, Maria Elisa Giuliani, Tariq Fellous, Alessandra Gentile, Marianna Carbone, Adele Cutignano, Laura Grauso, Laura Magliozzi, Gianluca Polese, Biagio D’Aniello, Fanny Defranoux, Serena Felline, Antonio Terlizzi, Antonio Calignano, Francesco Regoli, Vincenzo Di Marzo, Pietro Amodeo, Ernesto Mollo

**Affiliations:** 1 Institute of Biomolecular Chemistry, National Research Council of Italy, 80078 Pozzuoli, Italy; rmvitale@icb.cnr.it (R.M.V.); enrico.daniello@szn.it (E.D.); a.martella@lacdr.leidenuniv.nl (A.M.); cristoforo.silvestri.1@ulaval.ca (C.S.); tariq_fellous@hotmail.com (T.F.); alessandra.gentile@mpi-bn.mpg.de (A.G.); mcarbone@icb.cnr.it (M.C.); acutignano@icb.cnr.it (A.C.); lagrauso@unina.it (L.G.); f.defranoux@icb.cnr.it (F.D.); vdimarzo@icb.cnr.it (V.D.M.); 2 Department of Biology and Evolution of Marine Organisms, Stazione Zoologica Anton Dohrn, 80121 Naples, Italy; aterlizzi@units.it; 3 Department of Life and Environmental Sciences, Polytechnic University of Marche, 60131 Ancona, Italy; s.gorbi@univpm.it (S.G.); m.e.giuliani@univpm.it (M.E.G.); f.regoli@univpm.it (F.R.); 4 Faculty of Medicine, Laval University, Quebec, QC G1V 0A6, Canada; 5 Department of Agriculture, University of Naples “Federico II”, 80055 Portici, Italy; 6 Department of Biology, University of Naples “Federico II”, 80126 Naples, Italy; laura.magliozzi@unina.it (L.M.); gianluca.polese@unina.it (G.P.); biagio.daniello@unina.it (B.D.); 7 The National Interuniversity Consortium For Marine Sciences (CoNISMa), 00198 Rome, Italy; felline@conisma.it; 8 Department of Life sciences, University of Trieste, 34128 Trieste, Italy; 9 Department of Pharmacy, University of Naples “Federico II”, 80131 Naples, Italy; calignan@unina.it

**Keywords:** biological invasions, Mediterranean, *Caulerpa cylindracea*, caulerpin, molecular interactions, PPAR

## Abstract

Although the chemical warfare between invasive and native species has become a central problem in invasion biology, the molecular mechanisms by which bioactive metabolites from invasive pests influence local communities remain poorly characterized. This study demonstrates that the alkaloid caulerpin (CAU)—a bioactive component of the green alga *Caulerpa cylindracea* that has invaded the entire Mediterranean basin—is an agonist of peroxisome proliferator-activated receptors (PPARs). Our interdisciplinary study started with the in silico prediction of the ligand-protein interaction, which was then validated by in vivo, ex vivo and in vitro assays. On the basis of these results, we candidate CAU as a causal factor of the metabolic and behavioural disorders observed in *Diplodus sargus*, a native edible fish of high ecological and commercial relevance, feeding on *C. cylindracea*. Moreover, given the considerable interest in PPAR activators for the treatment of relevant human diseases, our findings are also discussed in terms of a possible nutraceutical/pharmacological valorisation of the invasive algal biomasses, supporting an innovative strategy for conserving biodiversity as an alternative to unrealistic campaigns for the eradication of invasive pests.

## 1. Introduction

Biological invasions are a main component of global change with profound ecological, economic and social consequences [1]. They are also of great concern for marine ecosystems [2], particularly for the Mediterranean Sea, where dense carpets of invasive algae are transforming vast coastal areas into alarming monocultures. Furthermore, recent research has emphasized the urgency of investigating metabolites produced by invasive plants and animals as stress factors in marine environments [3,4,5]. Since the pioneering efforts of Ernesto Fattorusso [6,7], both marine macrophytes and dinoflagellates are especially known to contain several bioactive natural products of ecological and ecotoxicological interest [8,9]. In this respect, crucial questions have been raised by some of the worst invasive macroalgae in the Mediterranean Sea, belonging to the genus *Caulerpa* [4,5,10,11,12]. Among them, *C. cylindracea* (previously known as *C. racemosa* var. *cylindracea*), which is native to South-western Australia [8], has become widely diffused in the whole Mediterranean, with profound consequences for native species resulting in significant ecological and economical costs. Currently, this invasive alga represents an important food item for the white sea bream *Diplodus sargus* (family: Sparidae), which is both a keystone species playing a major role in controlling the abundance of sea urchins in the Mediterranean [13,14,15], and a commercial species largely appreciated for human nutrition [11]. 

**Figure 1:**
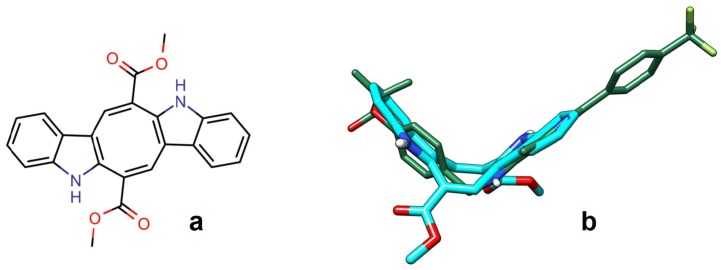
Structural comparison between CAU and a selective PPARα agonist. (**a**) CAU 2D structure and (**b**) a 3D fit of CAU (thick sticks, carbon = cyan) on GW590735 (thin sticks, carbon = green), a potent and selective PPARα agonist, showing the “V-shaped” structure shared by the two molecules.

The novel diet of white sea bream (see supplementary Video S1 showing a juvenile specimen of *D. sargus* swallowing the invasive alga with evident voracity) is attracting the increasing interest of researchers and local media since it correlates with metabolic disorders in the fish and with a decrease of essential lipids in its flesh [11,12,16,17,18]. The altered lipid metabolism of the fish could be explained by a combination of factors, including low levels of essential fatty acids in *C. cylindracea* and the possible role of algal metabolites as regulators of lipid metabolism in fish [11,12]. It is noteworthy that crude *C. racemosa* extracts turned out to also have dyslipidaemic effects in rats [19]. However, the molecular interactions between bioactive metabolites from *Caulerpa* species and their macromolecular targets responsible for the observed effects have remained unclear. In *D. sargus*, it has been shown that a *Caulerpa*-based diet alters the activity/expression of several proteins involved in lipid metabolism, including peroxisome proliferator-activated receptor alpha (PPARα), whose gene transcription was enhanced [12]. Moreover, there is an intriguing connection with other studies showing a decrease in both palatability and nutritional values in edible fish exposed to environmental concentrations of fenofibrate (FFB), a PPARα agonist with a recognized ecotoxicological potential. FFB, commonly used as a lipid-lowering drug in humans, induces lipid metabolism abnormalities and decreases EPA and DHA content when administered to freshwater fish (rainbow trout and grass carp) [20,21]. It is worthy to note that the same effect has been observed in wild populations of *D. sargus* feeding on *C. cylindracea* [11]. This observation is consistent with the PPARα-mediated transcription of genes related to hepatic β-oxidation, such as carnitine palmitoyltransferase 1 (CPT1) [22]. Remarkably, the algal secondary metabolite caulerpin (CAU, Figure 1a), which accumulates in the tissues of *D. sargus* and other edible Mediterranean fish species [11,12,16,17,18,23], shows several functional similarities to FFB since both compounds: (i) suppress activation of hypoxia-inducible factor-1 (HIF-1) [24,25], (ii) inhibit protein tyrosine phophatase-1B (PTP1B) [26,27] and (iii) show anti-inflammatory properties [28,29]. In addition, the PPARα-mediated effects of FFB on the social interaction of mice [30] can be paralleled to the ability of CAU of influencing the social behaviour in *D. sargus* [31]. However, despite considerable past efforts to identify the molecular targets of CAU, its possible direct interactions with PPARα, the main target of FFB, has not yet been investigated. The same applies to the possible interaction of CAU with the PPARγ isoform, which is also expressed in brain areas involved in regulation of behavioural processes in vertebrates [32,33]. We thus tested here if CAU, although exhibiting a totally different molecular scaffold from classical PPAR ligands, could modulate PPARα/γ transcriptional activity by direct ligand binding. This hypothesis was first addressed by *in silico* studies to predict the possible interaction of CAU with both the α and γ PPAR isoforms and then validated by luciferase reporter assays. Furthermore, the functional similarities between CAU and FFB have been deeply investigated by in vivo, ex vivo and in vitro experiments coupled with transcriptional analysis of genes of the PPARα signalling pathway.

## 2. Results

### 2.1. Computational Studies

By applying a ligand-based approach, we found that the rigid and V shaped bis-indolic structure of CAU (Figure 1a) shows a good overall fit with the bioactive conformations of classical PPARs ligands, an example of which is shown in Figure 1b; however, none of the CAU carboxymethyl-groups overlaps the carboxylic functional group occurring in the reference ligands.

**Figure 2:**
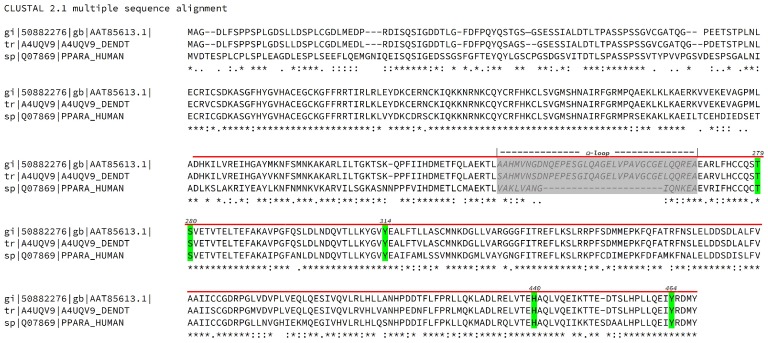
Multi-alignment of *Sparus aurata* (50882276), *Dentex dentex* (A4UQV9) and human (Q07869) PPARα sequences obtained with ClustalW program (CLUSTAL 2.1 multiple sequence alignment). Critical residues involved in binding with canonical agonists are depicted with a green background and numbered according to the human sequence. The Ligand Binding Domain (LBD) is indicated with a bold red line over the sequence. The Ω loop is indicated over the sequences, printed in medium grey and depicted with light grey background. The symbols under the sequences stand for sequence identity (*), strict conservation of residue type (:), loose conservation of residue type (.) and sequence variability (space).

These contrasting features definitively called for a target-based approach to check if the overall scaffold, including the orientation of its functional groups, was compatible with a PPARα and/or PPARγ agonist activity. Since such an approach needed a three-dimensional structure for PPAR targets and currently only partial PPARs sequences from fish of the family Sparidae are available, we decided to compare these sequences to the human ones, for which high-resolution reference structures are available. Results are shown in Figure 2.

As an example, a multi-alignment between two sparid fish (*Sparus aurata* and *Dentex dentex*) and human PPARα sequences (Figure 2) shows that both sparids share 68% of sequence identity and 80% of positives with the human protein in the Ligand Binding Domain (LBD) and that all the critical residues for agonist interaction are conserved among the species, the only relevant insertion being located in the flexible Ω-loop.

**Figure 3:**
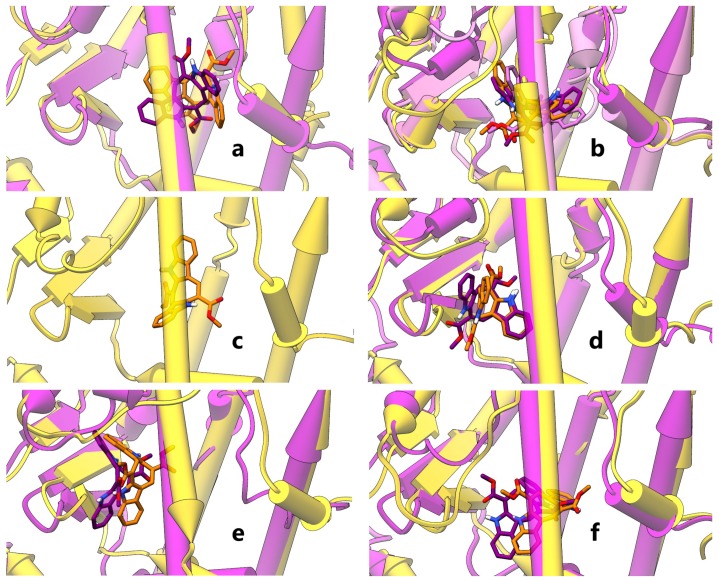
Representative MD structures of PPAR-CAU complexes. Panels a-f show CAU and representative MD structures of PPARα-CAU (magenta) and PPARγ-CAU (gold) complexes. In particular, panels show PPARα/γ I (**a**), PPARα II, III (this latter in mauve)/PPARγ II (**b**), PPARγ III (**c**), PPARα/γ IV (**d**), PPARα/γ V (**e**) and PPARα/γ VI (**f**) complex structures from MD representative frames. Proteins and ligands are shown as half-transparent pipes-and-planks and opaque stick with non-carbon atoms coloured by type (red: oxygen, blue: nitrogen, white: hydrogen), respectively. Complexes are grouped by similarity and shown in the same orientation, resulting from a recursive best-fit of protein heavy atoms, iterated by pruning distant atom pairs until no pair exceeds 2.0 Å.

Molecular docking studies followed by molecular dynamics (MD) simulations were thus undertaken for CAU on the crystallographic structures of both hPPARα and hPPARγ LBD, encompassing helices H3, H4-5, H7, H11, H12 and the β-sheet. The six representative poses, selected as described in the methods section, are shown in Figure 3. Three poses in PPARα (I–III, panels **a**,**b**) and two in PPARγ (I, II, panels **a**,**b**) embrace helix H3, while the ligand in PPARγ III pose runs almost parallel to helix H3 (panel c). One pose in both receptors (PPARα/γ IV, panel **d**) is located between helix H3 and β-sheet, one (PPARα/γ V, panel **e**) is oriented toward β-sheet and one (PPARα/γ VI, panel **f**), located between helices H3 and H7, embraces helix H5. In all the selected poses of both isoforms, CAU forms mainly hydrophobic ligand-protein interactions, enforced in some poses by H-bonds. In particular, for PPARα, CAU H-bonds with an occurrence >10% over MD production runs are detected with S280/T279 (pose II), I354CObb/T279 (III), Y334NHbb (IV) and S280/T279 (VI), where “bb” subscripts indicate protein backbone groups. In PPARγ complexes, CAU features intermolecular H-bonds with S342/R288 (V) and S289 (VI). The LDB region spanned by CAU poses overlaps with that of other PPARγ partial agonists in crystallographic structures, for example, magnolol [34], where two different orientations of the ligand occur in the LBD.

### 2.2. Luciferase Reporter Assays

**Figure 4:**
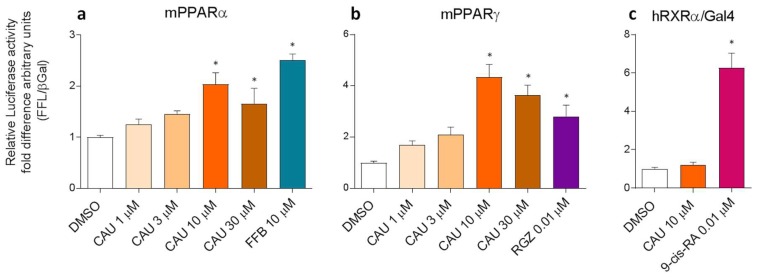
Luciferase reporter assays on COS7 cells. CAU activate mPPARα (**a**, *n* = 5) and mPPARγ (**b**, *n* = 6) but not RXRα (**c**, *n* = 9) in COS-7 cells. Fenofibrate (FFB), rosiglitazone (RGZ) and 9-cis retinoic acid (9-cis-RA) were used respectively as PPARα, PPARγ and RXRα agonist. Statistical analysis vs. vehicle (DMSO) were performed by one-way ANOVA, followed by Dunnett’s analysis. * *p*  <  0.05. Error bars indicate standard error of the mean.

The predicted dual PPARα/γ partial agonist behaviour of CAU was then validated by PPARα and PPARγ luciferase reporter assays on COS-7 cells, in comparison with FFB and rosiglitazone (RGZ), chosen as PPARα and PPARγ selective agonists, respectively (Figure 4a,b). The results show the activation of PPARα- and PPARγ-mediated transcription after exposure to CAU 10 µM and 30 µM, with levels comparable to those induced by FFB 10 µM and RGZ 0.01 µM. In addition, since luciferase assays were based on the co-expression of PPAR/RXR heterodimers, we also used the RXRα/Gal4 system to rule out the direct activation of RXR (Figure 4c) by CAU. The obtained results clearly supported a dual PPARα/γ partial agonist behaviour of CAU.

### 2.3. In Vivo Studies on D. sargus

The reported functional similarities between CAU and FFB led us to perform further investigations on the PPARα isoform, which is the main molecular target of FFB. In particular, a manipulative experiment allowed us to test the hypothesis that oral administration of purified CAU can influence PPARα gene expression in *D. sargus.*

**Figure 5:**
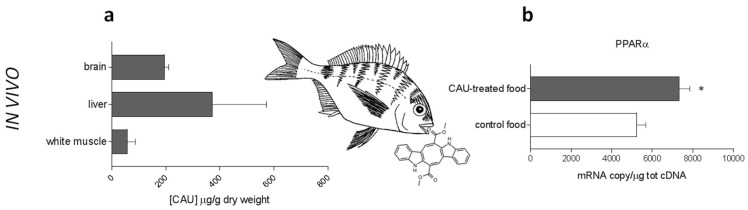
In vivo experiments. (**a**) CAU levels in the tissues of *D. sargus* fed with food treated with CAU (*n* = 4 for each bar). (**b**) PPARα gene expression in the liver of *D. sargus* fed with artificial food treated with purified CAU, in comparison with individuals fed with control food (*n* = 3 for each bar).

Juvenile fish were fed in controlled aquarium conditions for one month with CAU-enriched food. Subsequently, accumulation of CAU was evaluated in fish tissues (Figure 5a), showing that liver, brain and, to a lower extent, white muscle were able to accumulate orally-administered CAU. The measurement of relatively high levels of CAU in *D. sargus* brain provides the first evidence, to our knowledge, that the compound can cross the fish blood-brain barrier (BBB). Strikingly, accumulation of CAU in fish liver was paralleled by a significant upregulation of PPARα (Figure 5b), with induction levels comparable to those previously measured for *D. sargus* under field conditions [12].

### 2.4. Ex Vivo Studies on Precision-Cut Liver Slices (PCLS) of D. sargus and In Vitro Exposures of HepG2 Cells

**Figure 6:**
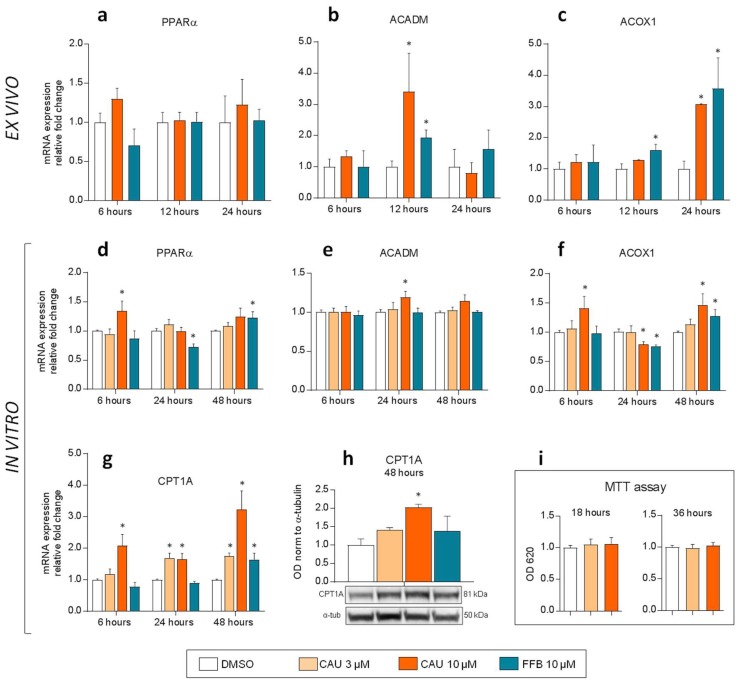
*Ex vivo* and in vitro experiments. (**a**–**c**) Time-dependent mRNA levels of PPARα, ACADM and ACOX1 under exposure of precision-cut tissue slices of *D. sargus* liver to either CAU or FFB (*n* = 3 for each bar). (**d**–**g**) Time-dependent expression of PPARα responsive genes in HepG2 cells. qPCR analyses were performed comparing PPARα (**d**), ACADM (**e**), ACOX1 (**f**) and CPT1A (**g**) gene expression relative to DMSO in cells treated with CAU and FFB at 6 h (*n* = 4), 24 h (*n* = 3) and 48 h (*n* = 4). (**h**) Expression of CPT1A protein in HepG2 cells (western blot) at 48 h (*n* = 2). Averaged optical density (OD) values for the CPT1A bands were normalized to that of α-tubulin. (**i**) MTT assays on HepG2 cells (*n* = 3). Statistical analyses were performed using unpaired *t*-tests. * *p*  <  0.05 vs. vehicle (DMSO). Error bars indicate standard error of the mean.

Experiments with PCLS of *D. sargus* confirmed similar trend in PPARα target gene induction by CAU and FFB (Figure 6a–c).

In particular, mRNA levels of ACADM increased after 12 h exposure to both CAU (3.4-fold) and FFB (1.9-fold) and ACOX1 showed a high up-regulation after 24 h exposure (3.0 and 3.6-fold for CAU and FFB, respectively). When assayed in a dose-dependent manner in a simpler cellular model (HepG2 cells), CAU and FFB induced the expression of PPARα responsive genes functioning in mitochondrial (ACADM, CPT1A) and peroxisomal (ACOX1) fatty acid β-oxidation (Figure 6d–g). In addition, we found significant overexpression of carnitine palmitoyltransferase 1A (CPT1A) protein at 48 h following CAU treatment in the same model (Figure 6h). The MTT viability assay demonstrated no cytotoxicity after exposure to CAU (Figure 6i).

## 3. Discussion

Although the increasing worldwide challenges raised by invasive species have attracted the attention of both biologists and global change researchers, it is still unclear what evolutionary responses should be expected in native species exposed to bioactive molecules never encountered before.

The activity and selectivity of bioactive compounds regulating fundamental processes such as development, reproduction, defense and nutrition, which sensibly affect ecosystem structure and stability, is mostly the result of long-term evolutionary trends. Conversely, compounds from invasive pests may start exerting unexpected and dramatic effects on the native communities, also playing a critical role in the behaviour, spread and impact of the invaders [4], thus significantly contributing to the rapid evolutionary changes characterizing biological invasions. 

In the present study, we focused on *D. sargus*, a fish with a fundamental ecological role in the Mediterranean, being the most important species involved in the sea-urchin cascade [14,15]. This fish also has substantial social and economic importance, representing a fishing target for direct human nutrition. Our previous investigations on this species revealed the onset of oxidative stress conditions related to the dietary accumulation of CAU, an alkaloid abundant in the green alga *C. cylindracea*, which is highly invasive in the Mediterranean and has become an important food item in the diet of *D. sargus*. In particular, the dietary exposure to *C. cylindracea* was shown to alter fatty acids metabolism and gene expression in *D. sargus* [12,23], acting as a relevant biotic stressor. However, the algal metabolites directly involved in the fish metabolic disorders were still unknown. 

In this report we candidate the interaction of CAU with PPARs, which are nuclear receptor proteins that function as transcription factors for genes involved in cellular differentiation, development, inflammation and lipid metabolism, as a possible cause of alterations in fish lipid metabolism.

In particular, we hypothesize and provide evidence for the direct binding and the activation by CAU of PPARα and PPARγ.

The functional analogies between CAU and the PPARα agonist FFB [24,25,26,27,28,29] led us to focus on the target genes of this PPAR isoform involved in fatty acid metabolism in tissues with high oxidative rates such as muscle, heart and liver. Some of such target genes, indeed, were upregulated by CAU in both the ex vivo and the in vitro model, supporting a role of CAU in the activation of lipid β-oxidation pathways. This explains, at least in part, the cellular and physiological alterations observed in fish eating *C. cylindracea*, which lead to a detrimental health status and altered behaviour, potentially preventing the reproductive success of fish populations [23].

Cascade effects of such molecular interactions still remain to be elucidated on the entire Mediterranean ecosystem, in which *D. sargus* is one of the main controllers of the abundance of major benthic grazers [35]. Nonetheless a PPARα mediated fat loss in *D. sargus* and other edible marine fish, similar to that induced by FFB on freshwater fish [20,21], would most likely produce a severe economic impact to the fishery sector. National newspapers have already reported a significant change in the organoleptic properties of *D. sargus* flesh as a possible consequence of the novel diet of based on *C. cylindracea* [36,37]. In addition, the interaction of CAU with PPARα and PPARγ, both expressed in brain areas involved in regulation of behavioural and emotional processes in rodents [30,32,33], could also result in PPAR-mediated altered social behaviour of *D. sargus*. Indeed, dietary CAU is already known to reduce the aggressive behaviour of *D. sargus* [31]. Since small molecules must cross the BBB to have an effect on centrally-mediated behaviours, the high levels of CAU here detected in the fish brain suggest that, in addition to the metabolic alterations discussed above, behavioural responses to the *C. cylindracea*-based diet could also have substantial impact over time on *D. sargus* population structure and ecology.

From a different perspective, however, the present study provides novel information that could help to turn the threat into an opportunity of combining sustainable development of the sea-based economy and conservation strategies. A first possible application of the results presented in this study could be the use of selected species of *Caulerpa* as functional foods. Feather-like species, such as *C. taxifolia* and *C. sertularioides*, contain high concentrations of the toxic sesquiterpene caulerpenyne (CYN) [4,5,6]. Conversely, other *Caulerpa* algae, collectively called “green caviar” or “sea grapes,” are widely exploited for human consumption in Indo-Pacific and Caribbean regions [38]: among these, *C. racemosa* and *C. lentillifera*, as well as *C. cylindracea*, are particularly suitable because of their low content in CYN [39,40], while they are rich in CAU [4,5,41]. Different opinions still persist on CAU toxicity [42] but the lack of acute toxicity assessed in the current study by in vitro toxicological tests at the relatively high maximum dose (10 µM) (Figure 6**i**), supports the hypothesis that the toxic symptoms observed in humans after ingestion of *Caulerpa* algae are most probably due to other algal components [43], including the toxic metabolite CYN. Sea grapes are mainly considered conventional foods [40] or generic dietary supplements for their fiber, anti-oxidant and other nutrients content [44]; their use as functional foods has been also considered based on possible antinociceptive and anti-inflammatory effects of CAU and sulphated polysaccharides [28,44,45]. The present study, by identifying CAU as a non-toxic PPAR agonist, opens up new perspectives for the exploitation of the invasive sea grape *C. cylindracea*, as a source of a novel functional ingredient potentially useful in the prevention of lifestyle-related diseases [46].

PPAR ligands are being considered also for their therapeutic potential in the control of metabolic disorders (i.e., hyperlipidaemia), atherosclerosis and for the treatment of the Alzheimer’s disease [47,48]. Given the situation of high uncertainty in the management of invasive pests in the Mediterranean Basin, the valorisation for pharmaceutical and nutraceutical purposes of undesired algal biomasses could propel their removal, thus helping to reduce the pressure of such invasive species [4]. 

Overall, our work demonstrates the validity of an interdisciplinary approach to delve deeper into molecular mechanisms of action through which specific natural substances from marine pests potentially exert cascade effects in the entire marine ecosystem, from molecular to progressively higher levels of biological complexity, up to fisheries-based economy. In addition, the identification of CAU as a PPAR agonist provides a suitable framework for mission-oriented projects that could make the control (i.e., the harvesting) of *C. cylindracea* a profitable and “virtuous” activity.

## 4. Materials and Methods 

### 4.1. Computational Studies

Computational methods have been utilized to compare the molecular structure of CAU with typical PPAR agonists and to predict and characterize its possible direct interaction with PPARα and PPARγ. Starting ligand geometry for ligand-based analysis and molecular docking was built with Ghemical 2.99.2 [49], followed by energy minimization (EM) at the molecular mechanics level first, using Tripos 5.2 force field parametrization [50] and then at the AM1 semi-empirical level. CAU was fully optimized using GAMESS [51] at the Hartree-Fock level with STO-3G basis set to derive the partial atomic charges using the RESP procedure of restrained fit to the HF/6-31G*/STO-3G electrostatic potential [52]. Docking studies were performed with AutoDock 4.2 [51]. Two crystallographic structures of both PPARα (PDB entry 2P54 and 1K7L) and PPARγ (PDB entry 2F4B and 4JAZ), complexed with different full agonists and the ligand were processed with AutoDock Tools (ADT) package version 1.5.6rc1 [53] to merge non polar hydrogens, calculate Gasteiger charges and select rotatable side-chain bonds. Grids for docking evaluation with a spacing of 0.375 Å and 70 × 60 × 70 points, centred in the ligand binding pocket, were generated using the program AutoGrid 4.2 included in Autodock 4.2 distribution. PPARα M330/M335, M330/I354/M355, M355/F318/Y314 and PPARγ M348/M364, M348/F363/M364, Y327/H449/M364 sidechains were selected as rotatable in different docking runs. One hundred molecular docking runs were performed with this setup: Lamarckian Genetic Algorithm (LGA), 100 individuals in a population with a maximum of 15 million energy evaluations and a maximum of 37,000 generations, followed by 300 iterations of Solis and Wets local search. The non-redundant representative poses from the most populated clusters within 1 Kcal/mol from the complex endowed with the most favourable binding energy were selected for the subsequent MD simulations of ligand-PPARα/γ complex. The complexes were completed by addition of all hydrogen atoms and underwent EM and then MD simulations with Amber16 pmemd.cuda module [54,55,56], using ff14SB version of AMBER force field [57] for the protein and gaff parameters [58] for the ligand. To perform molecular dynamics (MD) simulation in solvent, the complexes were confined in TIP3P water periodic truncated octahedron boxes exhibiting a minimum distance between solute and box surfaces of 10 Å, using the tleap module of AmberTools16 program [57]. The systems were then neutralized by addition of counterions (Na^+^) and underwent 1000 steps of EM with solute atoms harmonically restrained to their starting positions using a force constant of 10 kcal mol^−1^ Å^−1^. 90 ps restrained MD (5 kcal mol^−1^ Å^−1^) at constant volume was run on each solvated complex, gradually heating the system to 300 K, followed by 60 ps restrained MD (5 kcal mol^−1^ Å^−1^) at constant temperature (300 K) and pressure (1 atm) to adjust system density. Production MD simulations were carried out at constant temperature (300 K) and pressure (1 atm) for 50 ns, prolonged to 100 ns if rearrangements were observed during the first 50 ns, with a time-step of 2 fs. Bonds involving hydrogens were constrained using the SHAKE algorithm [59].

### 4.2. Extraction and Purification of CAU from C. cylindracea

CAU purification involved the extraction of *C. cylindracea* with acetone followed by partition between diethyl ether and water. The diethyl ether extract was subjected to silica gel and sephadex LH-20 column chromatography to give pure CAU (orange prisms) identified by comparison of ^1^H- and ^13^C-NMR recorded data in DMSO-*d*_6_ with the literature values [60,61]. NMR spectra (Figures S1–S2) were recorded on a 400 MHz Bruker Avance III HD spectrometer equipped with a CryoProbe Prodigy. Chemical shift values were referenced to the residual solvent peaks (δ*_H_* 2.49 ppm and δ*_C_* 39.5 ppm)

### 4.3. Luciferase Reporter Assays

Evidence for direct binding and activation of PPARs by CAU was provided by luciferase-based techniques. COS-7 cells (monkey kidney fibroblast-like cells) for PPARα and PPARγ luciferase reporter assays were grown in DMEM with l-Glutamine supplemented with 10% foetal bovine serum and 1% Pen/Strep under standard conditions. Cells were plated in a 24-well dishes at 70% confluence and transfected using Lipofectamine LTX and PLUS Reagent (Life Technologies, CA, USA: cat. no. 15338-100) according to the manufacture’s instruction. 

For the experiments on the CAU-mediated activation of PPARα and PPARγ, at day 1, for each well, a combination of 25 ng of mouse PSG5- PPARα (Addgene, MA, USA: cat. no. 22751) or mouse PPARγ1 (Addgene, MA, USA: cat. no. 8886), 300 ng of PPRE X3-TK-luc; (Addgene, MA, USA: cat. no. 1015), 100 ng of pSV-β-Galactosidase Control Vector (Promega, WI, USA: cat. no. E1081) and 75 ng pcDNA3 (Invitrogen, CA, USA) empty vector to a total of 500 ng were transfected. 

The next day, the growth media was replaced with fresh media containing CAU or positive controls in DMSO (vehicle) and treated overnight. In particular, fenofibrate 10 μM (FFB, Sigma Aldrich, MO, USA: cat. no. F6020) and rosiglitazone 0.01 μM (RGZ, Tocris Bioscience, MN, USA: cat. no. 5325,) were used respectively as PPARα and PPARγ agonists to be compared with CAU 1-30 μM. At day 3 after 18 h of treatment the cells were harvested and processed for the Luciferase and β-Galactosidase detection analysis. The Luciferase Gene Reporter activity was detected using the Luciferase kit (Sigma Aldrich, MO, USA: cat. no. LUC1) whereas the β-Galactosidase Activity was detected with the β-Galactosidase detection kit (Sigma Aldrich, MO, USA: cat. no. Gal-A). The β-Gal expression was quantified with the Microplate Readers (Tecan, Switzerland) and used as an internal experimental control to analyse the transfection efficiencies in each cell sample group. The levels of Firefly Luciferase chemiluminescence intensity was detected on a ChemiDoc MP system station using the Imagelab software (Bio-Rad, CA, USA) and normalized with respect to the β-Gal expression. 

To rule out the possible direct activation of RXR by CAU, COS-7 cells were grown, plated and transfected as described above. Briefly, at day1, for each well, a combination of 25 ng of CMX-Gal4-hRXRα (kindly provided by Steven Kliewer); 300 ng of TK-MH100x4-Luc containing the UAS enhancer elements (kindly provided by Steven Kliewer); 100 ng of pSV-β-Galactosidase Control Vector (Promega, WI, USA: cat. no. E1081) and 75 ng pcDNA3 (Invitrogen, CA, USA) empty vector to a total of 500 ng were transfected. The next day, the growth media was replaced with fresh media containing 9-cis retinoic acid 0.01 μM (9-cis-RA, Sigma Aldrich, MO, USA: cat. no. R4643) as a RXRα agonist and treated overnight in comparison with CAU 10 μM. DMSO was used as vehicle. At day 3 after 18 h of treatment the cells were harvested and processed for the Luciferase and β-Galactosidase detection analysis. 

### 4.4. In Vivo Experiments on D. sargus

Animal handling and experimental procedures were approved by the University of Napoli “Federico II” ethical committee on animal experiments (ethical permit protocol n. 2013/0096061 of 10/31/2013). The studies were conducted at the animal facility of the Department of Biology, University of Napoli “Federico II,” Napoli (Italy). Juvenile *D. sargus* were caught using a narrow mesh net. 10 fishes were distributed into two 100 L glass aquaria (five fish each) in a close circuit with a 100 L sump setup holding a mechanical and biological filter, a protein skimmer and a zeolite reactor. One aquarium was used as control and one for testing CAU-treated food. The fish were kept at 12 h light/12 h dark cycle at the temperature of 18 °C. Fish were fed once per day with 1 g of dry food per tank. Control food was prepared by soaking 30 g of Tetra Discus food granules (Tetra) in 60 mL acetone and then evaporating the organic solvent under reduced pressure, while treated food was made in the same manner but after dissolving 33.0 mg of CAU in the acetone. The dose of administered CAU was ten times higher than that measured in *C. cylindracea*, to assess short time accumulation in fish tissues. After one month of treatment the fish were euthanatized with an overdose of MS222 (tricaine methane sulfonate) (>150 mg/L), dissected and tissues (livers, brains and white muscles) frozen in liquid nitrogen and stored at −80 °C before the analyses (quantification of CAU and quantitative PCR of PPARα).

### 4.5. Ex Vivo Exposures on Precision-Cut Liver Slices (PCLS) of D. sargus

Adults of *D. sargus* (27 ± 2 cm; 500 ± 150 g) were obtained from an aquaculture farm, acclimatized in 90 L tanks at 18 °C for at least 2 weeks and fed daily, with commercial food. Fish were anesthetized on ice and sacrificed by decapitation. The livers were rapidly excised, placed in cold (4 °C) Hank’s Balanced Salt Solution (HBSS: KCl 0.40 g/L, KH_2_PO_4_ 0.06 g/L, NaCl 18 g/L, Na_2_HPO_4_ 0.05 g/L, d-glucose 1 g/L, pH 7.4, supplemented with 1% penicillin/streptomycin antibiotics mix) and washed by a cold HBSS flow through the main vessels to remove the blood and preserve the tissue from warm ischemia. For preparation of the slices, the liver was divided into pieces of approximately 5 × 5 mm sectioning area, using a sterile blade. Each piece of tissue was embedded in an agarose gel cylinder (2.5% low melting point agarose, cooled under 30 °C), to facilitate the slicing procedure. 250 µm slices were cut using a Leica vibrating blade vibratome VT1200S (Leica, Wetzlar, Germany) with a razor blade at the following settings: speed 0.2 mm/s; amplitude 1.0 mm; knife angle 18°. The whole procedure was carried on in cold HBSS. The slices were placed in 12-well culture plates (two slices per well), with 1.5 mL L-15 Leibovitz’s medium, supplemented with 10% FBS, 1% pen/strep mix and NaCl to a final concentration 18 g/L (L-15+). Slices were incubated on an orbital shaker at 18 °C under normal atmosphere. A 30 min pre-incubation was performed to remove any residual dead cells due to slicing. After the pre-incubation period, PCLS were exposed to CAU (purified as described in Section 2.2) and FFB dissolved in DMSO and used at a final 10 µM concentration in L-15+ medium. A solvent control plate with DMSO in L-15+ was also included. Exposed and control slices were cut from the same liver and incubated in parallel. After 6 h, 12 h and 24 h exposure, the slices were pooled (4 slices), frozen in liquid nitrogen and stored at −80 °C until subsequent RNA extraction and qPCR analyses. The exposure experiment was replicated for three different individual fish (*n* = 3).

### 4.6. In Vitro Experiments on HepG2 Cells

HepG2 cells were grown in DMEM with l-Glutamine supplemented with 10% foetal bovine serum and 1% Pen/Strep under standard conditions. Cells were cultured in 6 well plate and treated with CAU (3 µM and 10 µM) and FFB (10 µM) for 6, 24 and 48 h. After treatment the cells were washed in PBX 1X and used for subsequent quantitative PCR analysis and Western Blot. 

### 4.7. LC-MS Quantification of CAU in D. sargus Tissues

Samples of fish tissues (livers, brains and white muscles) obtained from in vivo experiments on *D. sargus*, were lyophilized and exhaustively extracted with acetone followed by partition between ethyl acetate and water. Levels of CAU accumulation in liver, brain and white muscle tissue of each specimen were measured by ultraperformance liquid chromatography/mass spectrometry (UPLC-MS/MS) by using an API 3200 Triple Quadrupole mass spectrometer (SCIEX, Framingham, MA, USA), following previously described methodologies [12,17]. Before extraction, indoleacrylic acid methyl ester was added as an internal standard. UPLC-MS/MS analyses of biological replicates are reported as mean (±SE, *n* = 3) values in µg g^−1^ (Figure 5a).

### 4.8. Quantification of Transcript Levels by qPCR

For in vivo and ex vivo samples obtained from *D. sargus* exposure experiments, absolute quantitative real-time PCR (qPCR) was performed to determine mRNA levels of specific target genes: PPARα in livers of *D. sargus* treated with CAU in vivo; PPARα, ACADM (acyl CoA dehydrogenase medium chain) and ACOX1 (acyl CoA oxidase 1) in *D. sargus* PCLS treated with CAU and FFB ex vivo. Total RNA was purified with Trizol reagent (Invitrogen, CA, USA), according to the manufacturer’s protocol and quantified using Nano-Drop ND-1000 UV-Visible Spectrophotometer (NanoDrop Technologies, Wilmington, DE, USA). RNA quality was verified on an agarose-formaldehyde gel. Total cDNA was reverse transcribed from 1 µg of total RNA for each sample using combined oligo(dT) and random hexamer primers (iScript cDNA Synthesis Kit, Bio-Rad). qPCR was performed using SYBR green method in StepOnePlus® Real-Time PCR System (Applied Biosystems) with specific primer pairs: PPARα (HG003581—96 bp amplicon): TGAGGGAGATCCACGGAGCCT (Fwd), TGAACGGCTGCTTGCTGGTCT (Rev); ACOX1 (LT671670—110 bp amplicon): GCTGATGAAATATGCCAAGGTG (Fwd), ACTCGCCAACAATCATGGA (Rev); ACADM (LT671671—122 bp amplicon): GTGAAGATGGGCGATGAGTATG (Fwd), TTGCTGGTTGGACATTTAGGATC (Rev). Each 15 µL DNA amplification reaction contained 7.5 µL of SYBR Select Master Mix (Life Technologies), 5 µL of total cDNA (diluted 1:5) and 200 nM of each forward and reverse primers. The real-time PCR program included an enzyme activation step at 95 °C (2 min) and 40 cycles each composed by 15 s at 95 °C, 15 s at the annealing temperature (PPARα: 62 °C; ACOX1: 59 °C; ACADM: 65 °C) and 1 min at 72 °C. The specificity of target cDNA amplification was checked by including controls lacking cDNA template and by a melting analysis (95 °C for 1 min, 65 °C for 10 s and fluorescence detection at increasing temperature between 65 and 95 °C). For each target gene, serial dilutions of known amounts of plasmid containing the amplicon of interest were used as standards. Samples and standards were run in duplicate in the same run. Cycle threshold (Ct) values of unknown samples were converted into mRNA copy number interpolating the standard plot of Ct versus log copy number. 

For in vitro samples of HepG2 cells exposed to CAU and FFB, the cells were immersed in Trizol (Invitrogen, CA, USA) after 1 wash in PBX 1X. RNA was subsequently collected using Pure link RNA Mini Kit (Invitrogen, CA, USA). Extracted RNA was then treated with DNase-I (1 U/mL; Sigma-Aldrich, MO, USA) following the manufacturer’s instructions. RNA integrity was estimated on the 1% agarose gel. 1 µg of total RNA was reverse transcribed ISCRIPT RT SUPERMIX (Biorad, CA, USA: cat. no. 1708841) according to the manufacturers’ instructions. Quantitative PCR was carried out in a real-time PCR system CFX384 (Bio-Rad) using the Sso Advanced SYBR Green supermix (Bio-Rad, CA, USA: cat. no. 170-8842). Expression levels were standardized to RPL27 as reference gene and all the data were analysed using 2−ΔΔCt Livak Method by using the following specific forward and reverse primers: Human RPL27 (123 bp amplicon): ATCGCCAAGAGATCAAAGATAA (Fwd), TCTGAAGACATCCTTATTGACG (Rev); Human ACADM (114 bp amplicon): AAGCTACTTGTAGAGCACCAAGC (Fwd), ACGACCAGAATCAACCTCCC (Rev); Human CPT1A (136 bp amplicon): CTACACGGCCGATGTTACGA (Fwd), TGACGTACTCCCAAAGGTGG (Rev); Human ACOX1 (135 bp amplicon): AAGTATGCCCAGGTGAAGCC (Fwd), AATGGTGCACGCCTTAGACA (Rev); Human PPARα (137 bp amplicon): GCGAACGATTCGACTCAAGC (Fwd), AACGAATCGCGTTGTGTGAC (Rev).

### 4.9. Western Methods

HepG2 cells treated with different compounds for 48 h were further analysed through Western Blot for protein expression. Cells were washed two times in cold PBS and lysed with lysis solution (150 mM NaCl, 1 mM EDTA, pH 7.4, 10 mM Tris-HCl, pH 8, 1% SDS and protease inhibitors). Lysates were then placed on an orbital shaker and incubated at 4 °C for 30 min. Subsequently, lysates were centrifuged for 15 min at 13,000× g at 4 °C and the supernatants transferred into clear tubes and quantified by DC Protein Assay (Bio-Rad). Subsequently, the samples (80 µg of total protein) were incubated for 10 min in bolt buffer plus sample reducing agents (Thermo Fisher Scientific, MA, USA) and loaded on bis-tris plus precast polyacrylamide gel (4–12%, Thermo Fisher Scientific, MA, USA: cat. no. NW04120BOX) and then transferred to a PVDF membrane. Filters were incubated overnight at 4 °C with the rabbit monoclonal anti-CPT1A, 1-1000, (Cell Signalling Technology, MA, USA: cat. no. 12252) and mouse monoclonal anti-tubulin, 1-5000, ( Sigma–Aldrich, MO, USA: cat. no. T8203) that was used to check for equal protein loading. Reactive bands were detected by chemiluminescence (ECL or ECL-plus; Perkin-Elmer). Images densitometry were analysed by means of Image J software.

### 4.10. MTT Assays

For toxicological evaluations, HepG2 cells were grown in DMEM with L-Glutamine and 1% Pen/Strep under standard conditions without supplemented with 10% foetal bovine serum in 48-well dishes. After adhesion, cells were serum-deprived and treated with the desired concentrations of compounds for 18 h and 36 h (the absence of serum was maintained during the treatments). The ability of cells to reduce MTT provided an indication of the mitochondrial integrity and activity and has been interpreted as a measure of cell viability. Absorbance at 620 nm was read on a GENius- Pro 96/384 Multifunction Microplate Reader (GENios-Pro, Tecan, Milan, Italy). Compounds were dissolved in DMSO. Optical density values from vehicle treated cells were defined as 100% of MTT-reducing activity and the effects were measured as a % of the inhibition of the measures obtained with vehicle alone.

### 4.11. Statistical Analysis

Data sets were compared by use of unpaired *t*-tests or, if necessary, with one-way ANOVA, followed by the Dunette’s multiple comparison test using GraphPad Prism 7 software. Statistically significant differences were accepted when the *p* value was at least <0.05. Data were expressed as mean ± SEM of the given number of experiments (*n*).

## 5. Conclusions

This work represents a paradigmatic model in the study of biological invasions at the level of molecular interactions. In particular, it delves deeper into molecular mechanisms underpinning metabolic and behavioural disorders occurring in *D. sargus*, a native fish playing key ecological roles and representing a popular food item in the Mediterranean, feeding on the invasive alga *C. cylindracea*. Our results disclose unprecedented molecular interactions likely exerting cascade effects between different levels of complexity, from cell receptor modulation, through physiological effects, to ecosystems, up to the sea-based economy. They also pave the way for the valorisation of invasive biomasses through the development of functional foods for human nutrition and/or drugs for treating chronic diseases (e.g. diabetes, obesity, atherosclerosis), prevalent in developed societies. 

## Supplementary Materials

The following are available online at http://www.mdpi.com/1660-3397/16/11/431/s1, Video S1: *D. sargus* eating *C. cylindracea*, Figure S1: ^1^H NMR spectrum of CAU, Figure S2: ^13^C-NMR spectrum of CAU
