# Supplementary material for: Fishing for Targets of Alien Metabolites: A Novel Peroxisome Proliferator-Activated Receptor (PPAR) Agonist from a Marine Pest
Source: Mar Drugs. 2018 Nov 3;16(11):431. doi: 10.3390/md16110431 (PMC6267082; doi:10.3390/md16110431)

Supplementary Information

Figure S1. ^{1}H NMR Spectrum of CAU (DMSO-d_6).

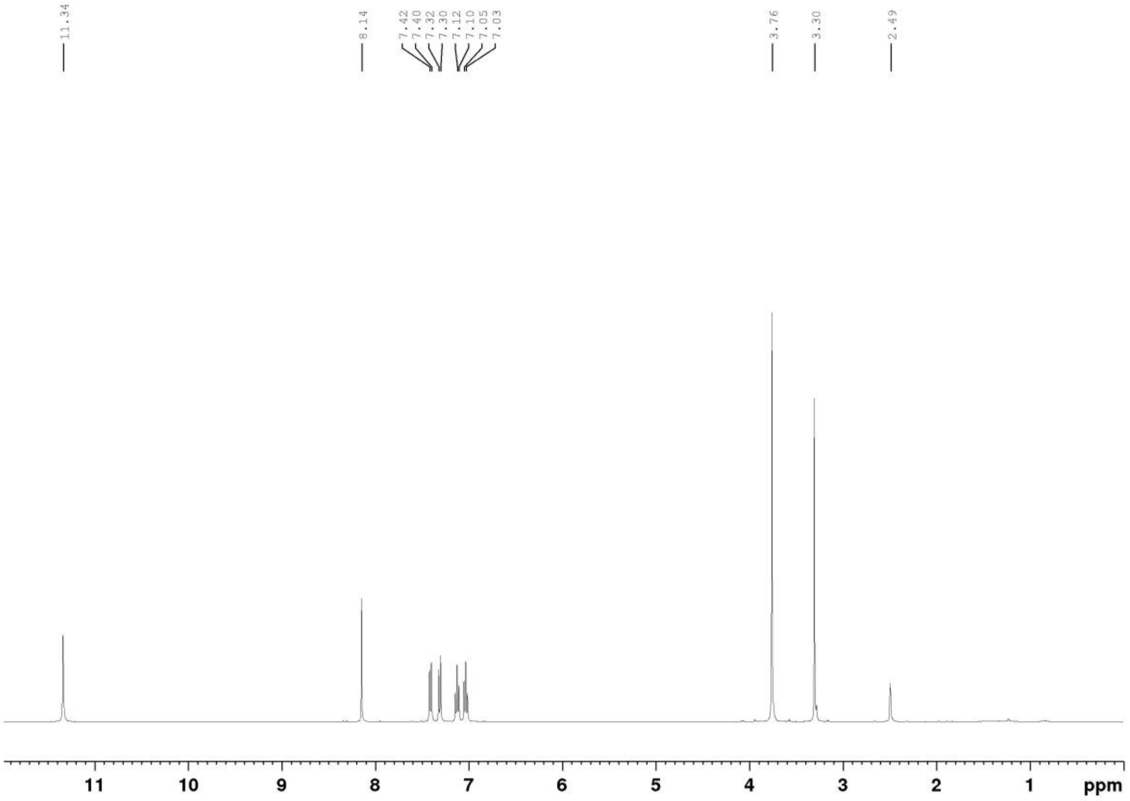

Figure S2. ^{13}C NMR Spectrum of CAU (DMSO-d_6).

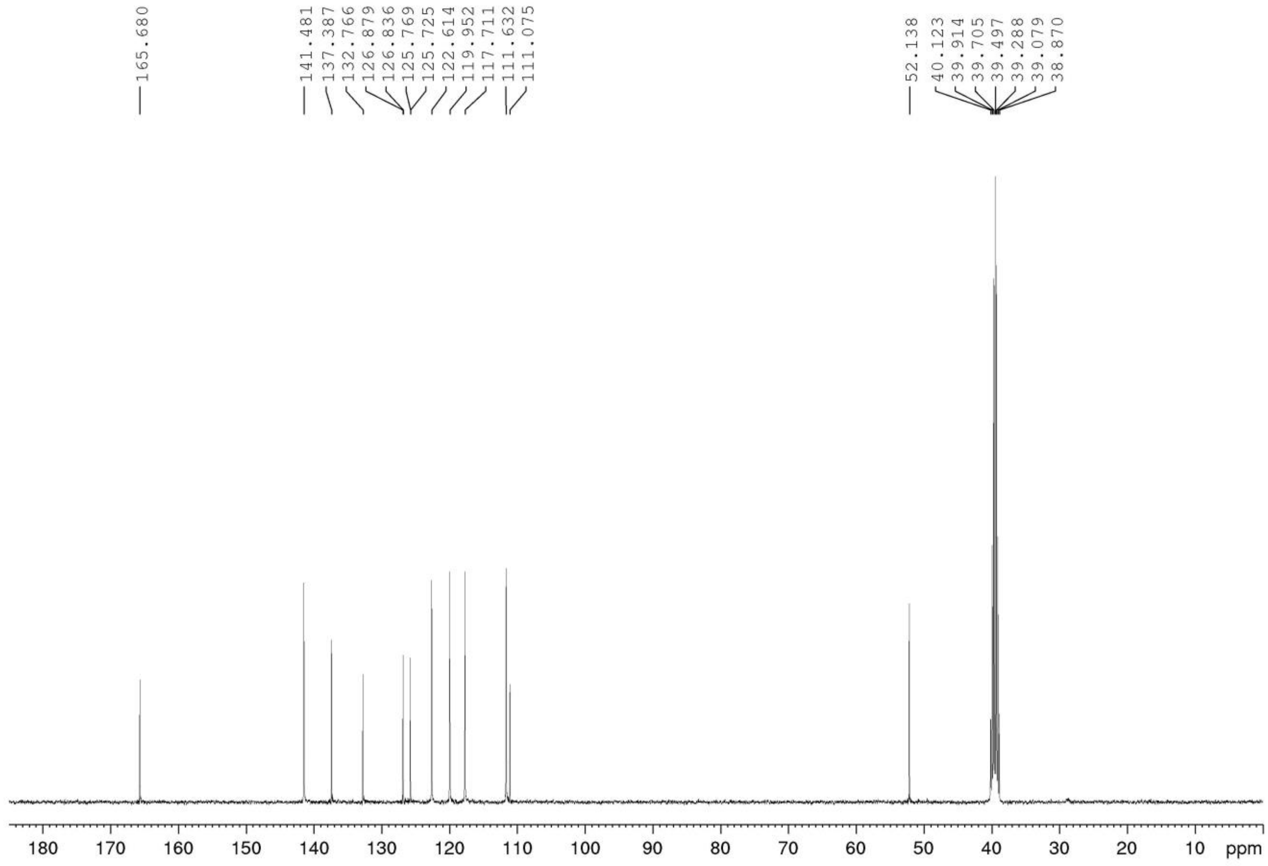

Supplement: Supplementary file 1 [file marinedrugs-16-00431-s001.zip › marinedrugs-382221-suppl.pdf]
